# Supplementary material for: Polyketide synthase gene clusters in the ericoid mycorrhizal fungus Oidiodendron maius: genome-wide prediction, expression profiling, and functional characterization of knockout mutants
Source: Front Plant Sci. 2026 Sep 16;17:1924430. doi: 10.3389/fpls.2026.1924430 (PMC13623903; doi:10.3389/fpls.2026.1924430)
Supplement: Supplementary file 1 [file SupplementaryFile1.zip › Supplementary Materials/Supplementary Folder 1.zip!/Oidma1_AssemblyScaffolds_Repeatmasked/knownclusterblast/cluster41/jgi.p_Oidma1_148724_mibig_hits.html]

| MIBiG Protein | Description | MIBiG Cluster | MiBiG Product | % ID | % Coverage | BLAST Score | E-value |
| --- | --- | --- | --- | --- | --- | --- | --- |
| EFY94505.1 | multidrug\_resistance\_protein | BGC0000337 | Destruxins\_biosynthetic\_gene\_cluster | 35 | 98.7529228371 | 714 | 1.9e-205 |
| AET14838.1 | multidrug\_resistance\_protein | BGC0001274\_c2 | Non-acetylated\_open-chain\_sophorolipid\_/\_diacetylated\_lactonic | 35 | 102.182385035 | 695 | 1.6e-199 |
| AAS92552.1 | SirA | BGC0001044 | Sirodesmin\_biosynthetic\_gene\_cluster | 36 | 98.9867498051 | 666 | 6.1e-191 |
| ctg1\_orf5 |  | BGC0000321 | Chaetocin\_biosynthetic\_gene\_cluster | 35 | 85.6586126267 | 582 | 1.2e-165 |
| XP\_011392729.1 | ABC\_transporter | BGC0001281 | Ustilagic\_acid\_biosynthetic\_gene\_cluster | 30 | 102.961808262 | 533 | 6.2e-151 |
| ACQ63630.1 | ABC\_multidrug\_resistance\_transporter | BGC0000875 | Caprazamycin\_biosynthetic\_gene\_cluster | 27 | 92.907248636 | 262 | 2e-69 |
| BAJ05895.1 | putative\_ABC\_multidrug\_resistance\_transporter | BGC0000872 | A-90289\_biosynthetic\_gene\_cluster | 26 | 94.2322681216 | 250 | 1e-65 |
| BAH43767.1 | ABC\_transporter\_ATP\_binding/permease\_protein | BGC0000452 | Tyrocidine\_biosynthetic\_gene\_cluster | 30 | 41.075604053 | 215 | 2.8e-55 |
| CAD89782.1 | hypothetical\_protein | BGC0001009 | Melithiazol\_biosynthetic\_gene\_cluster | 29 | 40.1402961808 | 214 | 8.3e-55 |
| AGL93176.1 | ABC\_transporter\_ATP-binding\_protein | BGC0000560 | Subtilomycin\_biosynthetic\_gene\_cluster | 28 | 46.5315666407 | 212 | 3.1e-54 |
| AGM16415.1 | paenibacterin\_ABC-transporter\_D | BGC0000400 | Paenibacterin\_biosynthetic\_gene\_cluster | 31 | 41.309431021 | 211 | 7e-54 |
| AFJ14797.1 | PlpH | BGC0000403 | Pelgipeptin\_biosynthetic\_gene\_cluster | 31 | 40.5300077942 | 206 | 1.7e-52 |
| AFJ14796.1 | PlpG | BGC0000403 | Pelgipeptin\_biosynthetic\_gene\_cluster | 32 | 41.3873733437 | 206 | 2.3e-52 |
| ACA97578.1 | PmxC | BGC0000408 | Polymyxin\_biosynthetic\_gene\_cluster | 32 | 41.5432579891 | 204 | 8.6e-52 |
| AEZ51518.1 | PmxC | BGC0001153 | Polymyxin\_biosynthetic\_gene\_cluster | 32 | 41.7770849571 | 203 | 1.1e-51 |
| AEP18658.1 | efflux\_transporter | BGC0000461 | WAP-8294A2\_(lotilibcin)\_biosynthetic\_gene\_cluster | 30 | 42.5565081839 | 201 | 4.2e-51 |
| BAE56595.1 |  | BGC0001123 | Aspirochlorine\_biosynthetic\_gene\_cluster | 35 | 29.9298519096 | 201 | 5.5e-51 |
| ACA97579.1 | PmxD | BGC0000408 | Polymyxin\_biosynthetic\_gene\_cluster | 30 | 45.6742010912 | 200 | 1.2e-50 |
| AEZ51519.1 | PmxD | BGC0001153 | Polymyxin\_biosynthetic\_gene\_cluster | 30 | 45.6742010912 | 200 | 1.2e-50 |
| CAD91219.1 | putative\_ABC\_transporter,\_Dbv24 | BGC0000289 | A40926\_biosynthetic\_gene\_cluster | 30 | 44.3491816056 | 200 | 1.2e-50 |
| AJM89737.1 | PmxD | BGC0001192 | Colistin\_biosynthetic\_gene\_cluster | 33 | 36.7108339829 | 200 | 1.6e-50 |
| AEH59056.1 | YvcC/ABC\_transporter | BGC0000385 | Lysobactin\_biosynthetic\_gene\_cluster | 30 | 40.3741231489 | 198 | 4.7e-50 |
| BAH43768.1 | ABC\_transporter\_ATP-binding/permease\_protein | BGC0000452 | Tyrocidine\_biosynthetic\_gene\_cluster | 30 | 45.1286048324 | 198 | 6.1e-50 |
| AJM89736.1 | PmxC | BGC0001192 | Colistin\_biosynthetic\_gene\_cluster | 32 | 41.6212003118 | 197 | 1e-49 |
| AJF34465.1 | ABC\_transporter | BGC0001207 | Teixobactin\_biosynthetic\_gene\_cluster | 28 | 42.4006235386 | 195 | 3e-49 |
| CAP20371.1 | ABC\_transporter\_ATP-binding\_membrane\_protein | BGC0000347 | Equibactin\_biosynthetic\_gene\_cluster | 29 | 46.9212782541 | 194 | 8.8e-49 |
| CAA90021.1 | PepT | BGC0000543 | Pep5\_biosynthetic\_gene\_cluster | 29 | 43.2579890881 | 193 | 1.5e-48 |
| ADX66465.1 | ScnB | BGC0000108 | Natamycin\_biosynthetic\_gene\_cluster | 29 | 41.3873733437 | 192 | 2.6e-48 |
| AAX31531.1 | putative\_ABC\_transporter | BGC0000336 | Daptomycin\_biosynthetic\_gene\_cluster | 31 | 37.2564302416 | 191 | 4.4e-48 |
| ABP54659.1 | ABC\_transporter\_related | BGC0000241 | Lomaiviticin\_biosynthetic\_gene\_cluster | 31 | 37.8020265004 | 190 | 9.8e-48 |
| ALK27913.1 | putative\_ABC\_transporter | BGC0001233 | Feglymycin\_biosynthetic\_gene\_cluster | 31 | 41.3873733437 | 189 | 2.2e-47 |
| AGM16416.1 | paenibacterin\_ABC-transporter\_E | BGC0000400 | Paenibacterin\_biosynthetic\_gene\_cluster | 28 | 47.9345284489 | 189 | 2.2e-47 |
| AAM54082.1 | ABC\_transporter | BGC0000020 | Ansamitocin\_biosynthetic\_gene\_cluster | 40 | 21.7459080281 | 188 | 3.7e-47 |
| CAC20925.1 | PimB\_protein | BGC0000125 | Pimaricin\_biosynthetic\_gene\_cluster | 29 | 40.9197194076 | 188 | 6.3e-47 |
| AFN69431.1 | ElxT | BGC0000509 | Epilancin\_15X\_biosynthetic\_gene\_cluster | 29 | 42.3226812159 | 187 | 8.3e-47 |
| AEH59102.1 | ABC\_transporter/permease/ATP-binding\_protein | BGC0000385 | Lysobactin\_biosynthetic\_gene\_cluster | 29 | 40.9976617303 | 185 | 3.1e-46 |
| CAG34715.1 | putative\_ABC\_transporter | BGC0000713 | Ribostamycin\_biosynthetic\_gene\_cluster | 30 | 43.8815276695 | 183 | 1.6e-45 |
| CAH58693.1 | putative\_exporter | BGC0000711 | Neomycin\_biosynthetic\_gene\_cluster | 30 | 38.1137957911 | 182 | 2.7e-45 |
| CAH05098.1 | ABC\_transport\_protein | BGC0000710 | Neomycin\_biosynthetic\_gene\_cluster | 30 | 38.1137957911 | 182 | 2.7e-45 |
| ABW74893.1 | ABC\_transporter | BGC0000759 | Diutan\_polysaccharide\_biosynthetic\_gene\_cluster | 29 | 43.3359314108 | 182 | 2.7e-45 |
| CAE53357.1 | ABC\_transporter | BGC0000440 | Teicoplanin\_biosynthetic\_gene\_cluster | 29 | 43.9594699922 | 182 | 2.7e-45 |
| CAG15016.1 | ABC\_transporter | BGC0000441 | Teicoplanin\_biosynthetic\_gene\_cluster | 29 | 43.9594699922 | 182 | 2.7e-45 |
| CAF33315.1 | putative\_ABC\_transporter,\_ATP-binding\_protein,\_NeoU | BGC0000709 | Neomycin\_biosynthetic\_gene\_cluster | 30 | 38.1137957911 | 182 | 2.7e-45 |
| CAI47637.1 | putative\_ABC-type\_aminoglycoside\_exporter | BGC0000692 | Apramycin\_biosynthetic\_gene\_cluster | 32 | 38.4255650818 | 182 | 3.5e-45 |
| CAG38700.1 | putative\_ABC-transporter,\_ATP-binding\_protein | BGC0000708 | Lividomycin\_biosynthetic\_gene\_cluster | 29 | 43.9594699922 | 181 | 4.5e-45 |
| AAP57696.1 | putative\_secretion\_protein | BGC0000768 | Gellan\_polysaccharide\_biosynthetic\_gene\_cluster | 28 | 43.2579890881 | 181 | 5.9e-45 |
| AHZ61877.1 | Lom43 | BGC0000240 | Lomaiviticin\_biosynthetic\_gene\_cluster | 29 | 38.2696804365 | 181 | 7.8e-45 |
| CAJ87588.1 | putative\_inner\_membrane\_ABC-transporter | BGC0001055 | Yersiniabactin\_biosynthetic\_gene\_cluster | 31 | 37.2564302416 | 181 | 7.8e-45 |
| ALE27500.1 | ABC\_transporter | BGC0001292 | A33853\_biosynthetic\_gene\_cluster | 31 | 37.412314887 | 181 | 7.8e-45 |
| AAF71763.1 | NysG | BGC0000115 | Nystatin\_biosynthetic\_gene\_cluster | 38 | 21.7459080281 | 180 | 1e-44 |
| AFD30962.1 | CrmT3 | BGC0000966 | Caerulomycin\_A\_biosynthetic\_gene\_cluster | 28 | 41.3873733437 | 180 | 1.3e-44 |
| CAP20370.1 | ABC\_transporter,\_ATP-binding\_membrane\_protein | BGC0000347 | Equibactin\_biosynthetic\_gene\_cluster | 28 | 44.8168355417 | 180 | 1.7e-44 |
| AAC44070.1 | unknown | BGC0000797 | Sphingan\_polysaccharide\_biosynthetic\_gene\_cluster | 28 | 43.2579890881 | 180 | 1.7e-44 |
| AGS77319.1 | ABC\_transporter | BGC0001178 | UK-68,597\_biosynthetic\_gene\_cluster | 29 | 45.2065471551 | 180 | 1.7e-44 |
| CCC55904.1 | putative\_ABC\_transporter,\_ATPase\_and\_permease\_component | BGC0000973 | Collismycin\_A\_biosynthetic\_gene\_cluster | 29 | 47.3109898675 | 179 | 2.3e-44 |
| AAK81823.1 | ABC\_transporter | BGC0000326 | Complestatin\_biosynthetic\_gene\_cluster | 28 | 43.4138737334 | 179 | 2.9e-44 |
| AFS60105.1 | PaenT | BGC0000540 | Paenibacillin\_biosynthetic\_gene\_cluster | 27 | 49.7272018706 | 178 | 3.8e-44 |
| AFD30963.1 | CrmT2 | BGC0000966 | Caerulomycin\_A\_biosynthetic\_gene\_cluster | 30 | 45.9080280592 | 178 | 5e-44 |
| ABQ84449.1 | salivaricin\_ABC-transporter | BGC0000624 | Salivaricin\_CRL1328\_alpha\_peptide\_/\_salivaricin\_CRL1328\_beta\_peptide | 27 | 46.687451286 | 178 | 5e-44 |
| EYT83450.1 | ABC\_transporter | BGC0001213 | Nataxazole\_biosynthetic\_gene\_cluster | 30 | 41.2314886984 | 178 | 6.6e-44 |
| CAA11793.1 | PCZA363.2 | BGC0000322 | Chloroeremomycin\_biosynthetic\_gene\_cluster | 28 | 43.9594699922 | 176 | 1.5e-43 |
| ACU62760.1 | ABC\_transporter\_related | BGC0001392 | Pinensins\_biosynthetic\_gene\_cluster | 37 | 21.7459080281 | 176 | 2.5e-43 |
| CCC55903.1 | putative\_ABC\_transporter,\_ATPase\_and\_permease\_component | BGC0000973 | Collismycin\_A\_biosynthetic\_gene\_cluster | 26 | 41.4653156664 | 175 | 3.3e-43 |
| AAM80540.1 | StaU | BGC0000290 | A47934\_biosynthetic\_gene\_cluster | 28 | 43.8815276695 | 175 | 4.3e-43 |
| CAC17506.1 | putative\_ABC\_transporter\_ATP-binding\_protein | BGC0000324 | Coelibactin\_biosynthetic\_gene\_cluster | 29 | 42.0109119252 | 175 | 4.3e-43 |
| AAK73498.1 | AmphG | BGC0000015 | Amphotericin\_biosynthetic\_gene\_cluster | 37 | 21.7459080281 | 175 | 5.6e-43 |
| CAG38699.1 | putative\_ABC-transporter,\_ATP-binding\_protein | BGC0000708 | Lividomycin\_biosynthetic\_gene\_cluster | 29 | 36.4770070148 | 174 | 7.3e-43 |
| WP\_009985592.1 | peptidase\_C39 | BGC0001311 | Flavecin\_biosynthetic\_gene\_cluster | 37 | 26.4224473889 | 173 | 1.6e-42 |
| CAB60262.1 | MrsT\_protein | BGC0000527 | Mersacidin\_biosynthetic\_gene\_cluster | 25 | 45.9080280592 | 173 | 1.6e-42 |
| EDY04298.1 | ABC\_transporter-related\_protein | BGC0000516 | Geobacillin\_II\_biosynthetic\_gene\_cluster | 29 | 40.3741231489 | 173 | 1.6e-42 |
| WP\_010071619.1 | ABC\_transporter\_ATP-binding\_protein | BGC0000578 | SRO15-2005\_biosynthetic\_gene\_cluster | 32 | 37.1784879189 | 173 | 2.1e-42 |
| CAF31837.1 | putative\_hygromycin\_B\_exporter | BGC0000699 | Hygromycin\_B\_biosynthetic\_gene\_cluster | 30 | 37.2564302416 | 173 | 2.1e-42 |
| ABS50189.1 | putative\_ABC\_transporter\_permease/ATP-binding\_protein | BGC0000410 | Pseudomonine\_biosynthetic\_gene\_cluster | 28 | 36.4770070148 | 171 | 4.7e-42 |
| AEI58864.1 | ABC\_transporter | BGC0000455 | Vancomycin\_biosynthetic\_gene\_cluster | 27 | 52.0654715511 | 171 | 4.7e-42 |
| CAG25756.1 | putative\_ABC\_transporter\_ATP-binding\_protein | BGC0000311 | Balhimycin\_biosynthetic\_gene\_cluster | 28 | 44.0374123149 | 171 | 6.1e-42 |
| AIS24845.1 | Dst21 | BGC0001147 | Distamycin\_biosynthetic\_gene\_cluster | 39 | 21.9017926734 | 171 | 8e-42 |
| EEM14530.1 | hypothetical\_protein | BGC0001210 | Pseudomycoicidin\_biosynthetic\_gene\_cluster | 28 | 44.8947778644 | 170 | 1e-41 |
| AIE77056.1 | ABC-transporter | BGC0000418 | Ristocetin\_biosynthetic\_gene\_cluster | 28 | 46.5315666407 | 170 | 1e-41 |
| KIH99790.1 | hypothetical\_protein | BGC0001176 | Streptomonomicin\_biosynthetic\_gene\_cluster | 30 | 43.5697583788 | 170 | 1.4e-41 |
| CAD15511.1 | probable\_composite\_atp-binding\_transmembrane\_abc\_transporter\_protein | BGC0001014 | Micacocidin\_biosynthetic\_gene\_cluster | 32 | 35.46375682 | 170 | 1.8e-41 |
| ABV83218.1 | CppG | BGC0000116 | Nystatin-like\_Pseudonocardia\_polyene\_biosynthetic\_gene\_cluster | 36 | 21.7459080281 | 169 | 3e-41 |
| ABD65953.1 | ABC\_transporter | BGC0000341 | Enduracidin\_biosynthetic\_gene\_cluster | 27 | 44.3491816056 | 168 | 5.2e-41 |
| ABS50188.1 | putative\_ABC\_transporter\_permease/ATP-binding\_protein | BGC0000410 | Pseudomonine\_biosynthetic\_gene\_cluster | 29 | 42.1667965705 | 168 | 5.2e-41 |
| CAD15510.1 | probable\_composite\_atp-binding\_transmembrane\_abc\_transporter\_protein | BGC0001014 | Micacocidin\_biosynthetic\_gene\_cluster | 27 | 42.0109119252 | 168 | 6.8e-41 |
| CAF32377.1 | putative\_ABC\_transporter,\_ATP-binding\_protein | BGC0000712 | Paromomycin\_biosynthetic\_gene\_cluster | 30 | 35.6196414653 | 167 | 1.2e-40 |
| CAJ87587.1 | putative\_ABC\_transporter\_protein | BGC0001055 | Yersiniabactin\_biosynthetic\_gene\_cluster | 30 | 34.6063912705 | 166 | 2e-40 |
| BAI23328.1 | putative\_ABC-transporter | BGC0000949 | A-500359s\_biosynthetic\_gene\_cluster | 29 | 39.5167575994 | 166 | 2.6e-40 |
| ALV82355.1 | ABC\_transporter | BGC0001370 | Lipopeptide\_8D1-1\_/\_lipopeptide\_8D1-2\_biosynthetic\_gene\_cluster | 39 | 22.2915042868 | 166 | 2.6e-40 |
| CAJ88629.1 | putative\_netropsin\_resistance\_protein\_subunit\_2 | BGC0000327 | Congocidine\_biosynthetic\_gene\_cluster | 29 | 38.4255650818 | 165 | 4.4e-40 |
| AAY93395.1 | 2-phenylethylamine\_uptake\_protein\_PeaH | BGC0000413 | Pyoverdine\_biosynthetic\_gene\_cluster | 28 | 42.8682774747 | 165 | 4.4e-40 |
| AIG79244.1 | Conserved\_putative\_membrane\_protein | BGC0000419 | Ristomycin\_A\_biosynthetic\_gene\_cluster | 28 | 46.453624318 | 164 | 9.8e-40 |
| BAJ19058.1 | putative\_ABC\_transporter | BGC0000288 | A-503083\_biosynthetic\_gene\_cluster | 37 | 22.2135619641 | 164 | 9.8e-40 |
| CAB38879.1 | putative\_ABC\_transporter | BGC0000315 | Calcium-dependent\_antibiotic\_biosynthetic\_gene\_cluster | 30 | 42.9462197973 | 163 | 1.7e-39 |
| BAP05577.1 | CalU | BGC0000967 | Calyculin\_biosynthetic\_gene\_cluster | 29 | 39.127045986 | 163 | 1.7e-39 |
| AFY23032.1 | TamA | BGC0000274 | Tetarimycin\_biosynthetic\_gene\_cluster | 38 | 20.1870615744 | 163 | 1.7e-39 |
| ACP43452.1 | McnB | BGC0000590 | Microcin\_N\_biosynthetic\_gene\_cluster | 28 | 39.360872954 | 163 | 2.2e-39 |
| CAJ44959.1 | MchF\_protein | BGC0000587 | Microcin\_H47\_biosynthetic\_gene\_cluster | 29 | 40.841777085 | 162 | 2.9e-39 |
| BAJ19059.1 | putative\_ABC\_transporter | BGC0000288 | A-503083\_biosynthetic\_gene\_cluster | 29 | 39.0491036633 | 162 | 3.7e-39 |
| AIS24846.1 | Dst20 | BGC0001147 | Distamycin\_biosynthetic\_gene\_cluster | 33 | 25.7989088075 | 162 | 3.7e-39 |
| CAE55703.1 | probable\_microcin\_H47\_secretion\_ATP-binding\_protein\_mchF | BGC0000589 | Microcin\_M\_biosynthetic\_gene\_cluster | 29 | 40.841777085 | 161 | 4.9e-39 |
| CAF32376.1 | putative\_ABC\_transporter,\_ATP-binding\_protein | BGC0000712 | Paromomycin\_biosynthetic\_gene\_cluster | 29 | 40.5300077942 | 161 | 8.3e-39 |
| CAG34716.1 | putative\_ABC\_transporter | BGC0000713 | Ribostamycin\_biosynthetic\_gene\_cluster | 28 | 39.9064692128 | 160 | 1.1e-38 |
| EFI65097.1 | lantibiotic\_transport\_protein | BGC0001407 | Bicereucin\_biosynthetic\_gene\_cluster | 28 | 43.9594699922 | 160 | 1.8e-38 |
| WP\_043270371.1 | ABC\_transporter\_ATPase | BGC0001188 | Legonaridin\_biosynthetic\_gene\_cluster | 27 | 45.8300857366 | 160 | 1.8e-38 |
| ADC96668.1 | ABC\_transporter | BGC0001076 | Liposidomycin\_biosynthetic\_gene\_cluster | 30 | 38.6593920499 | 160 | 1.8e-38 |
| ANP43732.1 | ThsT | BGC0001391 | Thusin\_biosynthetic\_gene\_cluster | 28 | 38.3476227592 | 160 | 1.8e-38 |
| BAB04170.1 | lantibiotic\_mersacidin\_transporter\_system | BGC0000517 | Haloduracin\_alpha\_/\_haloduracin\_beta\_biosynthetic\_gene\_cluster | 27 | 40.1402961808 | 160 | 1.8e-38 |
| EYT83451.1 | ABC\_transporter\_ATP-binding\_protein | BGC0001213 | Nataxazole\_biosynthetic\_gene\_cluster | 36 | 21.4341387373 | 159 | 3.2e-38 |
| CAF31838.1 | putative\_hygromycin\_B\_exporter | BGC0000699 | Hygromycin\_B\_biosynthetic\_gene\_cluster | 28 | 44.0374123149 | 158 | 4.1e-38 |
| CAI47638.1 | putative\_ABC-type\_aminoglycoside\_exporter | BGC0000692 | Apramycin\_biosynthetic\_gene\_cluster | 27 | 45.6742010912 | 158 | 5.4e-38 |
| AHJ59536.1 | ABC\_transporter\_protein | BGC0000502 | Cerecidin\_/\_cerecidin\_A1\_/\_cerecidin\_A2\_/\_cerecidin\_A3\_/\_cerecidin | 27 | 40.7638347623 | 158 | 5.4e-38 |
| CAC17507.1 | putative\_ABC\_transporter\_ATP-binding\_protein | BGC0000324 | Coelibactin\_biosynthetic\_gene\_cluster | 33 | 26.8121590023 | 158 | 7e-38 |
| BAI23327.1 | putative\_ABC-transporter | BGC0000949 | A-500359s\_biosynthetic\_gene\_cluster | 26 | 42.0109119252 | 156 | 2e-37 |
| ALE27499.1 | ABC\_transporter | BGC0001292 | A33853\_biosynthetic\_gene\_cluster | 26 | 45.7521434139 | 156 | 2e-37 |
| BAM09402.1 | putative\_ABC-transporter | BGC0000619 | Gassericin\_T\_biosynthetic\_gene\_cluster | 26 | 47.3109898675 | 155 | 5.9e-37 |
| ALX37944.1 | GaeT | BGC0001388 | Gassericin\_E\_biosynthetic\_gene\_cluster | 26 | 47.3109898675 | 154 | 7.8e-37 |
| AAL08400.1 | MceG | BGC0000586 | Microcin\_E492\_biosynthetic\_gene\_cluster | 28 | 39.9844115355 | 153 | 1.3e-36 |
| CAJ88628.1 | putative\_netropsin\_resistance\_protein\_subunit\_1 | BGC0000327 | Congocidine\_biosynthetic\_gene\_cluster | 28 | 36.1652377241 | 153 | 1.7e-36 |
| ABV83219.1 | CppH | BGC0000116 | Nystatin-like\_Pseudonocardia\_polyene\_biosynthetic\_gene\_cluster | 23 | 41.075604053 | 153 | 1.7e-36 |
| ADX66464.1 | ScnA | BGC0000108 | Natamycin\_biosynthetic\_gene\_cluster | 24 | 43.02416212 | 152 | 3e-36 |
| BAL15734.1 | ABC\_transporter | BGC0000432 | Streptothricin\_biosynthetic\_gene\_cluster | 29 | 45.1286048324 | 152 | 3e-36 |
| CAC20924.1 | PimA\_protein | BGC0000125 | Pimaricin\_biosynthetic\_gene\_cluster | 24 | 42.2447388932 | 152 | 3e-36 |
| EPE34342.1 | P-loop\_containing\_nucleoside\_triphosphate\_hydrolase | BGC0001035 | Pneumocandin\_biosynthetic\_gene\_cluster | 24 | 65.6274356976 | 151 | 6.6e-36 |
| AHH99931.1 | ABC\_transporter\_protein | BGC0000002 | Aculeximycin\_biosynthetic\_gene\_cluster | 27 | 42.3226812159 | 150 | 1.5e-35 |
| AAF71764.1 | NysH | BGC0000115 | Nystatin\_biosynthetic\_gene\_cluster | 27 | 40.2182385035 | 148 | 5.6e-35 |
| BAL72550.1 | lariatin\_biosynthetic\_protein | BGC0000575 | Lariatin\_biosynthetic\_gene\_cluster | 26 | 44.9727201871 | 146 | 2.1e-34 |
| AAK73499.1 | AmphH | BGC0000015 | Amphotericin\_biosynthetic\_gene\_cluster | 23 | 40.9976617303 | 146 | 2.8e-34 |
| AAG28766.1 | CoaD | BGC0000617 | Coagulin\_biosynthetic\_gene\_cluster | 25 | 47.7007014809 | 145 | 4.7e-34 |
| BAD72775.1 |  | BGC0000552 | Smb\_A\_/\_Smb\_B\_biosynthetic\_gene\_cluster | 27 | 41.7770849571 | 144 | 1.1e-33 |
| CBL93714.1 | ABC\_multidrug\_transporter | BGC0000360 | GE81112\_biosynthetic\_gene\_cluster | 27 | 38.5035074045 | 139 | 2e-32 |
| BAE07080.1 | possible\_ABC\_transporter | BGC0000693 | Butirosin\_biosynthetic\_gene\_cluster | 27 | 47.1551052221 | 137 | 1.3e-31 |
| ABL74935.1 | TlmT | BGC0001048 | Tallysomycin\_biosynthetic\_gene\_cluster | 26 | 43.5697583788 | 136 | 2.9e-31 |
| AHW81482.1 | hypothetical\_protein | BGC0000934 | Endophenazines\_biosynthetic\_gene\_cluster | 30 | 26.1886204209 | 135 | 3.7e-31 |
| BAU50927.1 | ABC\_transporter | BGC0001379 | BD-12\_biosynthetic\_gene\_cluster | 26 | 39.127045986 | 134 | 1.1e-30 |
| AMM63163.1 | AniL | BGC0001371 | Echinocandin\_B\_biosynthetic\_gene\_cluster | 35 | 21.8238503507 | 132 | 4.1e-30 |
| AFT91383.1 | EcdL | BGC0000338 | Echinocandin\_B\_biosynthetic\_gene\_cluster | 32 | 23.1488698363 | 131 | 9.2e-30 |
| AAK27372.1 | ABC\_transporter | BGC0000623 | Plantaricin\_W\_alpha\_/\_plantaricin\_W\_beta\_biosynthetic\_gene\_cluster | 25 | 35.3078721746 | 130 | 1.6e-29 |
| AHF21240.1 | CrnT | BGC0000500 | Carnolysin\_A1\_/\_carnolysin\_A2\_biosynthetic\_gene\_cluster | 26 | 39.360872954 | 128 | 7.8e-29 |
| AHF21235.1 | PabT | BGC0000542 | Paenicidin\_B\_biosynthetic\_gene\_cluster | 25 | 38.5814497272 | 127 | 1e-28 |
| AEK64492.1 | EtnT | BGC0000506 | Entianin\_biosynthetic\_gene\_cluster | 28 | 25.4871395168 | 124 | 1.1e-27 |
| AAD56148.1 | MutT | BGC0000533 | Mutacin\_III\_biosynthetic\_gene\_cluster | 24 | 39.9064692128 | 124 | 1.1e-27 |
| AAB91587.1 | SpaT | BGC0000559 | Subtilin\_biosynthetic\_gene\_cluster | 28 | 25.4871395168 | 121 | 5.6e-27 |
| EMC15128.1 | ABC\_transporter,\_ATP-binding\_and\_permease\_protein | BGC0000530 | Mutacin\_B-Ny266\_biosynthetic\_gene\_cluster | 25 | 39.6726422447 | 121 | 9.5e-27 |
| BAP82652.1 | ABC\_transporter | BGC0001148 | Pheganomycin\_biosynthetic\_gene\_cluster | 28 | 41.8550272798 | 119 | 3.6e-26 |
| CRG85577.1 | hypothetical\_protein | BGC0001402 | Cyclochlorotine\_biosynthetic\_gene\_cluster | 25 | 41.309431021 | 113 | 1.5e-24 |
| WP\_003950017.1 | ABC\_transporter\_ATP-binding\_protein | BGC0000546 | SAL-2242\_biosynthetic\_gene\_cluster | 34 | 20.5767731878 | 113 | 2e-24 |
| AAL15565.1 | putative\_SpaT | BGC0000511 | Ericin\_A\_biosynthetic\_gene\_cluster | 24 | 46.687451286 | 113 | 2.6e-24 |
| CAA19962.1 | putative\_ABC\_transporter | BGC0000551 | SapB\_biosynthetic\_gene\_cluster | 32 | 24.2400623539 | 112 | 4.4e-24 |
| ANQ31705.1 | ABC\_transporter | BGC0001395 | Teichomycin\_biosynthetic\_gene\_cluster | 26 | 27.7474668745 | 110 | 1.3e-23 |
| CBG75489.1 | putative\_ABC-transport\_system\_ATP-binding\_component | BGC0000423 | Scabichelin\_biosynthetic\_gene\_cluster | 29 | 23.6944660951 | 109 | 2.9e-23 |
| CBG75488.1 | putative\_ABC\_transport\_system\_integral\_membrane\_component | BGC0000423 | Scabichelin\_biosynthetic\_gene\_cluster | 24 | 40.2182385035 | 108 | 8.3e-23 |
| AAC63532.1 | sublancin\_transporter\_protein | BGC0000558 | Sublancin\_168\_biosynthetic\_gene\_cluster | 23 | 49.4154325799 | 108 | 8.3e-23 |
| AEG64672.1 | ABC\_transporter-like\_protein | BGC0000379 | Laspartomycin\_biosynthetic\_gene\_cluster | 31 | 20.9664848012 | 107 | 1.4e-22 |
| WP\_020635024.1 | multidrug\_ABC\_transporter\_ATPase | BGC0001211 | Albachelin\_biosynthetic\_gene\_cluster | 26 | 41.6991426345 | 106 | 1.9e-22 |
| CAM02315.1 | putative\_ABC\_transporter\_transmembrane | BGC0000349 | Erythrochelin\_biosynthetic\_gene\_cluster | 23 | 40.9197194076 | 98 | 6.6e-20 |
| CAB53321.1 | putative\_ABC\_transporter\_transmembrane\_protein | BGC0000325 | Coelichelin\_biosynthetic\_gene\_cluster | 25 | 21.278254092 | 97 | 8.6e-20 |
| ACU73106.1 | ABC\_transporter\_related | BGC0000501 | Catenulipeptin\_biosynthetic\_gene\_cluster | 24 | 38.6593920499 | 96 | 3.3e-19 |
| AAX31527.1 | putative\_ABC\_transporter | BGC0000336 | Daptomycin\_biosynthetic\_gene\_cluster | 28 | 23.3826968044 | 95 | 4.3e-19 |
| CAM02316.1 | ABC\_transporter\_protein,\_ATP-binding\_component | BGC0000349 | Erythrochelin\_biosynthetic\_gene\_cluster | 23 | 42.4785658613 | 94 | 9.5e-19 |
| BAC87901.1 | probable\_ferric\_acinetobactin\_transport\_system\_ATP-binding\_protein | BGC0000294 | Acinetobactin\_biosynthetic\_gene\_cluster | 26 | 20.7326578332 | 91 | 8.1e-18 |
| EWM63070.1 | ABC\_transporter\_ATP-binding\_protein | BGC0000679 | Diazepinomicin\_biosynthetic\_gene\_cluster | 21 | 36.6328916602 | 89 | 2.4e-17 |
| CAX48975.1 | ABC-transporter | BGC0000519 | Labyrinthopeptin\_A1,A3\_/\_labyrinthopeptin\_A2\_biosynthetic\_gene | 30 | 22.992985191 | 89 | 4e-17 |
| CAH94305.1 | putative\_ABC\_transporter\_StrV | BGC0000717 | Streptomycin\_biosynthetic\_gene\_cluster | 24 | 37.4902572097 | 87 | 1.5e-16 |
| BAG22744.1 | putative\_ABC-type\_multidrug\_transport\_system\_ATPase\_and\_permease\_component | BGC0000724 | Streptomycin\_biosynthetic\_gene\_cluster | 24 | 37.4902572097 | 87 | 1.5e-16 |
| ACU73107.1 | ABC\_transporter\_related | BGC0000501 | Catenulipeptin\_biosynthetic\_gene\_cluster | 25 | 40.4520654716 | 85 | 4.4e-16 |
| AEK75511.1 | ATP-binding\_ABC\_peptide\_transporter | BGC0000001 | Abyssomicin\_biosynthetic\_gene\_cluster | 27 | 20.9664848012 | 77 | 1.2e-13 |
| AAG32539.1 | SalX | BGC0000548 | Salivaricin\_A\_biosynthetic\_gene\_cluster | 25 | 20.2650038971 | 76 | 3.5e-13 |
| ACN39026.1 | putative\_UvrA-drug\_resistance\_pump | BGC0000448 | Tomaymycin\_biosynthetic\_gene\_cluster | 27 | 21.1223694466 | 69 | 4.3e-11 |
| AHI59113.1 | ATPase | BGC0001005 | Locillomycin\_biosynthetic\_gene\_cluster | 25 | 21.3561964147 | 66 | 2.1e-10 |
| AAK33690.1 | putative\_ABC\_transporter\_(ATP-binding\_protein) | BGC0000566 | Streptolysin\_S\_biosynthetic\_gene\_cluster | 26 | 20.4208885425 | 65 | 6.2e-10 |
| CAF31834.1 | hypotheticl\_protein | BGC0000699 | Hygromycin\_B\_biosynthetic\_gene\_cluster | 22 | 22.9150428683 | 64 | 8.1e-10 |
| CCJ67643.1 | cyclic\_peptide\_transporter;\_siderophore\_ABC\_transporter | BGC0001127 | Jagaricin\_biosynthetic\_gene\_cluster | 22 | 34.4505066251 | 58 | 7.6e-08 |
| AFO93372.1 | ABC\_transporter,\_ATP-binding\_protein | BGC0001362 | Tropodithietic\_acid\_biosynthetic\_gene\_cluster | 21 | 21.9017926734 | 57 | 1.3e-07 |
